# Vectorial capacity and *TEP1* genotypes of *Anopheles gambiae* sensu lato mosquitoes on the Kenyan coast

**DOI:** 10.1186/s13071-022-05491-5

**Published:** 2022-12-01

**Authors:** Brian Bartilol, Donwilliams Omuoyo, Jonathan Karisa, Kelly Ominde, Charles Mbogo, Joseph Mwangangi, Marta Maia, Martin Kibet Rono

**Affiliations:** 1 Kenya Medical Research Institute, Centre for Geographic Medicine Research-Coast, Kilifi, Kenya; 2 Pwani University Bioscience Research Centre (PUBReC), Pwani University, Kilifi, Kenya; 3 Centre for Tropical Medicine and Global Health, Nuffield Department of Medicine, University of Oxford, Old Road Campus Roosevelt Drive, Oxford, OX3 7FZ UK

**Keywords:** *Anopheles merus*, Thioester-containing protein 1, Allele, Kenya

## Abstract

**Background:**

Malaria remains one of the most important infectious diseases in sub-Saharan Africa, responsible for approximately 228 million cases and 602,000 deaths in 2020. In this region, malaria transmission is driven mainly by mosquitoes of the *Anopheles gambiae* and, more recently, *Anopheles funestus* complex. The gains made in malaria control are threatened by insecticide resistance and behavioural plasticity among these vectors. This, therefore, calls for the development of alternative approaches such as malaria transmission-blocking vaccines or gene drive systems. The thioester-containing protein 1 (*TEP1*) gene, which mediates the killing of *Plasmodium falciparum* in the mosquito midgut, has recently been identified as a promising target for gene drive systems. Here we investigated the frequency and distribution of *TEP1* alleles in wild-caught malaria vectors on the Kenyan coast.

**Methods:**

Mosquitoes were collected using CDC light traps both indoors and outdoors from 20 houses in Garithe village, along the Kenyan coast. The mosquitoes were dissected, and the different parts were used to determine their species, blood meal source, and sporozoite status. The data were analysed and visualised using the R (v 4.0.1) and STATA (v 17.0).

**Results:**

A total of 18,802 mosquitoes were collected, consisting of 77.8% (*n* = 14,631) *Culex* spp., 21.4% (*n* = 4026) *An. gambiae* sensu lato, 0.4% (*n* = 67) *An. funestus*, and 0.4% (*n* = 78) other *Anopheles* (*An. coustani*, *An. pharoensis*, and *An. pretoriensis*). Mosquitoes collected were predominantly exophilic, with the outdoor catches being higher across all the species: *Culex* spp. 93% (IRR = 11.6, 95% Cl [5.9–22.9] *P* < 0.001), *An. gambiae* s.l. 92% (IRR = 7.2, 95% Cl [3.6–14.5]; *P* < 0.001), *An. funestus* 91% (IRR = 10.3, 95% Cl [3.3–32.3]; *P* < 0.001). A subset of randomly selected *An. gambiae* s.l. (*n* = 518) was identified by polymerase chain reaction (PCR), among which 77.2% were *An. merus*, 22% were *An. arabiensis*, and the rest were not identified. We were also keen on identifying and describing the *TEP1* genotypes of these mosquitoes, especially the *R3/R3 allele that was identified recently in the study area. We identified the following genotypes among *An. merus*: *R2/R2, *R3/R3, *R3/S2, *S1/S1, and *S2/S2. Among *An. arabiensis*, we identified *R2/R2, *S1/S1, and *S2/S2. Tests on haplotype diversity showed that the most diverse allele was TEP1*S1, followed by TEP1*R2. Tajima’s *D* values were positive for TEP1*S1, indicating that there is a balancing selection, negative for TEP1*R2, indicating there is a recent selective sweep, and as for TEP1*R3, there was no evidence of selection. Phylogenetic analysis showed two distinct clades: refractory and susceptible alleles.

**Conclusions:**

We find that the malaria vectors *An. gambiae* s.l. and *An. funestus* are predominantly exophilic. *TEP1* genotyping for *An. merus* revealed five allelic combinations, namely *R2/R2, *R3/R3, *R3/S2, *S1/S1 and *S2/S2, while in *An. arabiensis* we only identified three allelic combinations: *R2/R2, *S1/S1, and *S2/S2. The TEP1*R3 allele was restricted to only *An. merus* among these sympatric mosquito species, and we find that there is no evidence of recombination or selection in this allele.

**Graphical Abstract:**

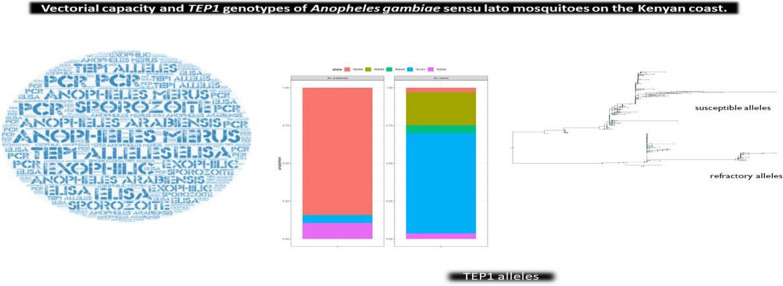

**Supplementary Information:**

The online version contains supplementary material available at 10.1186/s13071-022-05491-5.

## Background

Malaria remains one of the most important infectious diseases in sub-Saharan Africa, responsible for approximately 228 million cases and 602,000 deaths in 2020 [1]. Significant advances in malaria control have been achieved in the last two decades, mostly by vector control interventions including long-lasting insecticide-treated nets (LLINs) and indoor residual spraying (IRS). Between 2000 and 2019 we saw a reduction of 28% and 44% in global malaria incidence and mortality, respectively [2, 3]. Unfortunately, this progress has plateaued in the last 5 years, and the World Health Organization (WHO)’s 2016–2030 global technical strategy for malaria (GTS) [4] is off-track, with a global incidence reduction of less than 2% between 2015 and 2020 [1].

Malaria transmission in sub-Saharan Africa is mainly driven by mosquitoes of the *Anopheles gambiae* and, more recently, *Anopheles funestus* complexes [5–7]. Despite the scale-up of malaria control interventions, insecticide resistance [6, 8] and increased vector behavioural plasticity [9–13] are threatening the gains made in malaria control. This, therefore, calls for the development of more effective novel interventions such as transmission-blocking vaccines or gene drive systems that can lead to population replacement of infection-susceptible mosquitoes with those that are refractory to *Plasmodium* spp. infection [14].

Although there are 475 *Anopheles* species, only 70 are known primary or secondary vectors of malaria [15]. This is partly attributable to the mosquito’s innate immunity against invading parasites [16, 17]. One such anti-parasitic immunity is mediated by thioester-containing protein 1 (*TEP1*), a homologue of the mammalian complement factor 3. *TEP1* recognises and binds to ookinetes, mediating parasite lysis and melanisation [8]. *TEP1* is a highly polymorphic protein consisting of variants differentiated by amino acid sequence variation in the thioester domain (TED) region [18]. The variants are classified into two main subclasses: refractory TEP1*R (*R1 and *R2) and susceptible TEP1*S (*S1 and *S2). Mosquitoes bearing the R alleles are more effective at parasite killing, with *R1/R1 and *S2/S2 mosquitoes being fully resistant and susceptible to infection, respectively [16]. The TEP1*S and TEP1*R alleles are found in all species of *An. gambiae* s.l., albeit with marked variation in distribution both geographically and within the different species. Additionally, *TEP1* alleles have been shown to affect male fitness in wild mosquito populations, and due to their ability to clear off defective sperms, mosquitoes bearing the TEP1*S2 alleles have been shown to have higher fertility rates [19].

Recently, a novel resistance-encoding allele, termed TEP1*R3, was discovered in *Anopheles merus* populations from the Kenyan coast [20]. Its genetic diversity and functional role in controlling the development of malaria parasites within the mosquito have not been described. The present study aimed at characterising the *TEP1* alleles of *An. merus* populations collected from coastal Kenya.

## Methods

### Study area

Entomological collections were conducted in Garithe village, Kilifi County, along the Kenyan coast (Additional file 1: Fig. S1). This region is highly diverse, made up of dense forests, dry thorny bushes, savannah vegetation, and seasonal swamps of brackish water. There are two distinct rainy seasons: long rains which occur between April and July, and short rains between October and November [21]. The site was targeted for collections based on previous studies that described *An. merus* in high density [22].

Mosquitoes were collected in 20 houses over six consecutive days during the month of November in 2019 (Additional file 1: Fig. S1). Collections were carried out using Centers for Disease Control and Prevention light traps (CDC-LT) both indoors and outdoors for each house from dusk (1800 h) to dawn (0600 h), whilst coordinates were collected using eTrex^®^ 10 (Garmin, Kansas, United States of America). The indoor traps were set in houses where at least one person spent the night during the collection period. The outdoor traps were strategically set next to the livestock shed, and where livestock were absent, the trap was set approximately 5 m from the household selected for indoor sampling. The collected mosquitoes were identified morphologically in the field laboratory [23] and sorted by physiological stage and sex. All the *Anopheles* spp. were preserved individually in 1.5 ml microcentrifuge tubes containing silica pellets and transported to the KEMRI Wellcome Trust Research Programme (KWTRP) laboratory and stored at −80 °C.

### Mosquito processing

Using sterile scalpel and forceps, the female *Anopheles* mosquitoes were dissected and separated into distinct body parts for different assays. The legs and wings were used for *An. gambiae* s.l. sibling species identification, the head and thorax for the *Plasmodium falciparum* infection status, and the abdomen of blood-fed mosquitoes for trophic pattern and preference analysis as described previously [24].

### *Anopheles gambiae* s.l. sibling species identification

Genomic deoxyribonucleic acid (DNA) was extracted from the legs and wings of mosquitoes as described previously, with minor modifications [25]. Briefly, the mosquito parts were transferred into 1.5 ml microcentrifuge tubes containing 50 µl of 20% Chelex and crushed using polypropylene pestles. The lysate was incubated at 100 °C while shaking at 650 revolutions per minute (rpm) using a ThermoMixer (Eppendorf, Hamburg, Germany). The solution was then centrifuged at 10,000×*g* for 2 min, and the supernatant was transferred to a new 1.5 ml microcentrifuge tube. This was repeated twice, and the DNA was stored at −80 °C.

*Anopheles gambiae* s.l. sibling species were identified using a previously described method using primers that target the intergenic spacer (IGS) region of the ribosomal DNA [26]. The species were distinguished by their band sizes after running agarose gel electrophoresis as follows: 153 base pairs (bp) for *Anopheles quadriannulatus*, 315 bp for *An. arabiensis*, 390 bp for *An. gambiae* sensu stricto, 464 bp for *Anopheles melas*, and 466 bp for *An. merus* [26]. *Anopheles funestus* complex sibling species were also identified using polymerase chain reaction (PCR) with primers targeting the internal transcribed spacer region 2 (ITS2) [27].

### Blood meal analysis

The abdomens of the blood-fed female *Anopheles* mosquitoes were crushed in 50 µl of molecular-grade water using sterile polypropylene pestles. Thirty microlitres of the lysate was mixed with 500 µl of phosphate-buffered saline (PBS) and then used to determine the source of blood meal using direct enzyme-linked immunosorbent assays (ELISA) as described previously [28, 29], with slight modifications. The samples were tested against the anti-host immunoglobulin G (IgG): human, goat, bovine, and chicken. The results were read visually as described previously [30].

### *Plasmodium falciparum* sporozoite analysis

The mosquito head and thorax were crushed in 100 µl of 1× PBS in 1.5 ml microcentrifuge tubes. Then, 10 µl of 10× saponin was added to the lysate. The solution was subsequently incubated at room temperature for 20 min, and then centrifuged at 20,000×*g* for 2 min, and the supernatant was discarded. The pellet was resuspended in 100 µl 1× PBS. The solution was then centrifuged at 2000×*g* for 2 min and the supernatant was discarded. The pellet was resuspended in 50 µl of 20% Chelex and DNA extracted using the procedure described above. Thereafter, the extracted DNA was used for SYBR Green real-time PCR (RT-PCR) assays using primers described in Hermsen et al. [31]. Briefly, the RT-PCR reaction consisted of 7.5 µl of QuantiTect SYBR Green PCR master mix (Qiagen, Hilden, Germany), 7.5 µM of forward primer 5′-GTAATTGGAATGATAGGATTTACAAGGT-3′ and 7.5 µM of the reverse primer 5′-TCAACTACGAACGTTTTAACTGCAAC-3′, 2 µl of nuclease-free water, and 4 µl of the DNA. RT-PCR conditions were as follows: 95 °C for 10 min for HotStarTaq DNA Polymerase activation, 40 cycles of 95 °C for 30 s, 60 °C for 45 s, 68 °C for 45 s, and finally the melt curve phase: 95 °C for 15 s, 60 °C for 1 min, and 95 °C for 30 s.

### *TEP1* genotyping

The highly polymorphic TED region of *TEP1* was amplified using the primers VB229 5′-TCAACTTGGACATCAACAAGAAG-3′ and VB004 5′-ACATCAATTTGCTCCGAGTT-3′ as described previously [19, 32]. Thereafter, the 1088 ± 1-bp amplicon was cleaned using the QIAquick PCR purification kit (Qiagen, Hilden, Germany) and eluted in 15 µl of DNase-free water. The samples were subjected to both Sanger and next-generation sequencing (NGS).

For the Sanger sequencing, the PCR amplicons were sequenced using the primers (VB004 and VB229), BigDye Terminator chemistry v 3.1 (Applied Biosystems, UK), and the reaction was run on an ABI 3730xl capillary sequencer (Applied Biosystems, UK). The resulting sequence chromatograms were curated, edited, and aligned using the CLC Main Workbench 7 (CLC Bio, Qiagen, Aarhus, Denmark).

For NGS, 75 cycles of paired-end sequencing were carried out on the MiSeq platform using the Nextera DNA Flex library preparation protocol and MiSeq reagent kit v 3 (Illumina, USA). The resulting reads were demultiplexed, and then FastQC v 0.11.9 was used to remove indexes, low-quality PhiX and adapter reads. The identity of the resulting reads was ascertained using both BLASTn v 2.11.0 and mapping onto the *TEP1* references.

### Phylogenetic analysis

Multiple sequence alignment and sequence editing was conducted in AliView v 1.25 software, using the newly sequenced *TEP1-TED* sequences and those collated from GenBank [33]. Maximum likelihood phylogenies were reconstructed using the TIM+F+I substitution model with four gamma categories (TIM+F+I+G4) as the best fitting model as inferred by jModelTest in iqtree v 1.6.9 [34] and visualised using FigTree v 1.4.4.

### Statistical analysis

Statistical analysis, visualisation, and mapping were performed using R software (v 4.1.0) [35]. The data were further analysed using a generalised negative binomial regression model with a log link and robust errors, where the dependent variable was the number of mosquitoes collected, and the independent variables were site (indicator variable), day, and household using STATA v 17.0 (StataCorp, College Station, TX, USA). The human blood index (HBI) was calculated as the proportion of mosquitoes that had fed on humans divided by total blood meals tested for each species.

Haplotype statistics including the number of haplotypes, haplotype frequency, and haplotype configuration, and tests of natural selection including Tajima’s *D* [36], Fu and Li’s *D*, and Fu and Li’s *F* [37] were calculated using DNA Sequence Polymorphism (DnaSP) software [38].

## Results

### Abundance and diversity of *Anopheles* spp. mosquitoes

**Fig. 1:**
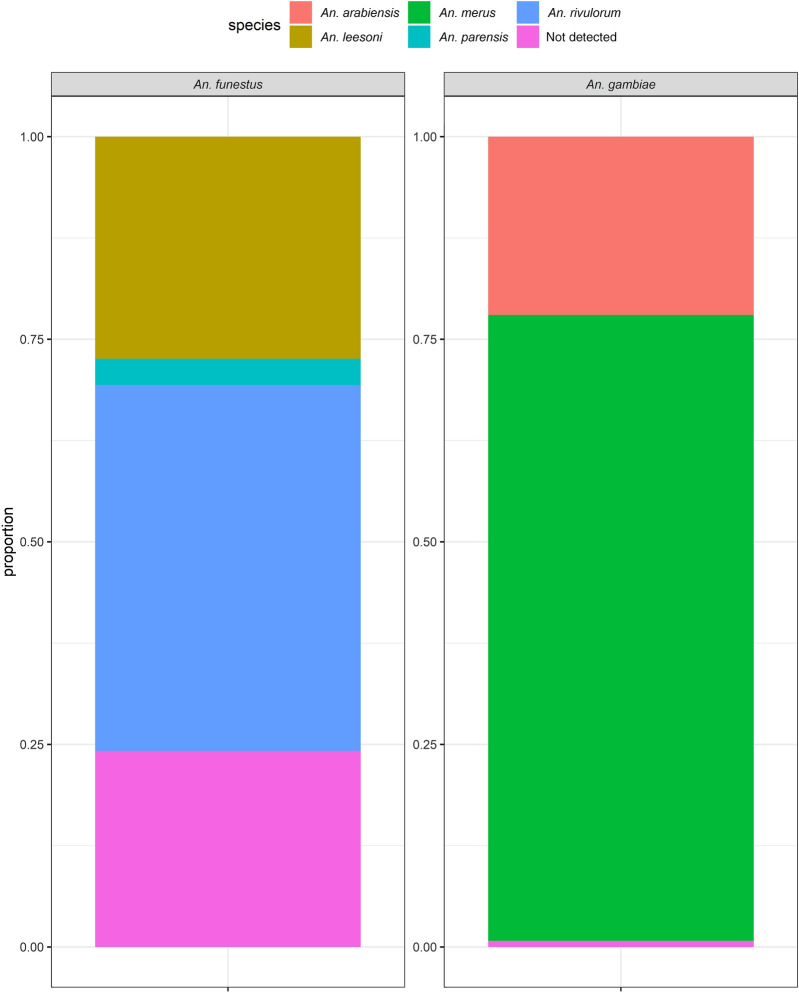
Proportion of the sibling species of *Anopheles gambiae* and *Anopheles funestus* complex identified by polymerase chain reaction

A total of 18,802 mosquitoes were collected. Based on morphological cues, the mosquito species consisted of 77.8% (*n* = 14,631) *Culex* spp., 21.4% (*n* = 4026) *An. gambiae* s.l., 0.4% (*n* = 67) *An. funestus*, and 0.4% (*n* = 78) other *Anopheles* (*An. coustani*, *An. pharoensis*, *An. pretoriensis*). A subset of the *Anopheles gambiae* s.l. (*n* = 518) mosquitos were analysed for sibling species by PCR. Of these, 77.2% (*n* = 400) were identified as *An. merus*, 22% (*n* = 114) as *An. arabiensis*, and 0.8% (*n* = 4) were not detected by PCR. This could be due to very low DNA concentrations, the presence of PCR inhibitors, or morphological misidentification (Fig. 1). *Anopheles funestus* complex consisted of 42% (*n* = 28) *An. rivulorum*, 25% (*n* = 17) *An. leesoni*, and 3% (*n* = 2) *An. parensis*, and the rest could not be detected (30%, *n* = 20).

A greater proportion of all the mosquito species collected were found outdoors (Table 1). Ninety-one percent of the *An. funestus* mosquitoes collected were caught outdoors (IRR = 10.3, 95% Cl [3.3–32.3]; *P* < 0.001). The same was observed for *An. gambiae* s.l., at 92% (IRR = 7.2, 95% Cl [3.6–14.5]; *P* < 0.001), and *Culex* spp. at 93% (IRR = 11.6, 95% Cl [5.9–22.9] *P* < 0.001).

**Table 1:** Comparisons of outdoor and indoor mosquitoes using negative binomial generalised linear models, where the dependent variable was the number of collected mosquitoes, and independent variables were site (indicator variable), day of collection, and household

Complex	Site	Number of sampling days	Number of households	No.	Median	IQR	IRR	IRR 95% CI	*P*-value
*An. funestus* complex	Indoors	6	20	6	0	[0–0]	1	–	–
	Outdoors	6	20	61	0	[0–2]	10.3	[3.3–32.3]	< 0.001
*An. gambiae* complex	Indoors	6	20	333	1	[0–6]	1	–	–
	Outdoors	6	20	3693	5	[0–51]	7.2	[3.6–14.5]	< 0.001
*Culex* spp.	Indoors	6	20	1076	13.5	[7–25]	1	–	–
	Outdoors	6	20	13,555	33.5	[10–200]	11.6	[5.9–22.9]	< 0.001

*IQR*, interquartile range, *IRR* incident rate ratio

Generally, *An. funestus*, *An. gambiae*, and *Culex* spp. are exophilic

### Physiological state and source of blood meal

Generally, there are a greater number of mosquitoes in all the physiological states found outdoors (Additional file 2: Table S1). Of those that were blood-fed, 90 mosquitoes belonged to the *An. gambiae* complex and five to the *An. funestus* complex. Those analysed for blood meal source consisted of 79% (*n* = 71) *An. merus* and 14.4% (*n* = 13) *An. arabiensis*. Among *An. merus* collected outdoors, a greater proportion had fed on goats (77.1%, *n* = 54), and 2.8% (*n* = 2) had fed on humans (HBI = 0.03). The HBI was however slightly higher indoors (HBI = 0.14). Among *An. arabiensis* collected outdoors, 38.5% (*n* = 5) had fed on goats and 23.1% on humans (*n* = 3, *HBI* = 0.27) (Table 2).

**Table 2:** Blood meal source identification both indoors and outdoors and estimates of the human blood index

Species	Location	Human	Bovine	Goat	Bovine and goat	Not detected	HBI
*An. arabiensis*	Indoor	0	0	1 (7.7%)	0	1 (7.7%)	N/A
	Outdoor	3 (23.1%)	1 (7.7%)	5 (38.5%)	1 (7.7%)	1 (7.7%)	0.27
*An. merus*	Indoor	1 (1.4%)	3 (4.3%)	2 (2.9%)	1 (1.4%)	0	0.14
	Outdoor	2 (2.8%)	2 (2.8%)	55 (77.5%)	1 (1.4%)	4 (5.7%)	0.03

### Plasmodium infection in mosquitoes

Of the 4026 *An. gambiae* s.l., 281 mosquitoes (*An. arabiensis* [*n* = 70] and *An. merus* [*n* = 211]) were screened for sporozoite infection by PCR (Additional file 2: Table S1). From these, 9.5% (20/211) *An. merus* and 8.6% (6/70) *An. arabiensis* were sporozoite-positive. Furthermore, a greater number of mosquitoes from outdoor catches were sporozoite-positive for both species (Additional file 3: Table S2).

### *TEP1* distribution

**Fig. 2:**
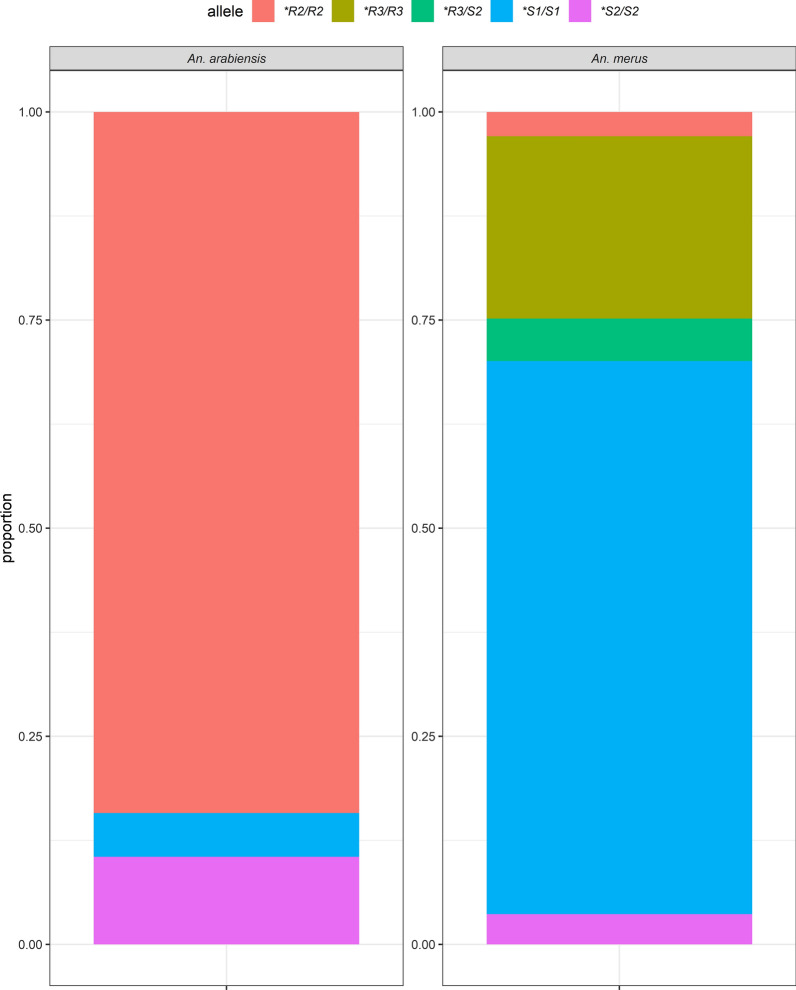
Proportion of the *TEP1* alleles among *Anopheles arabiensis* and *Anopheles merus*

**Fig. 3:**
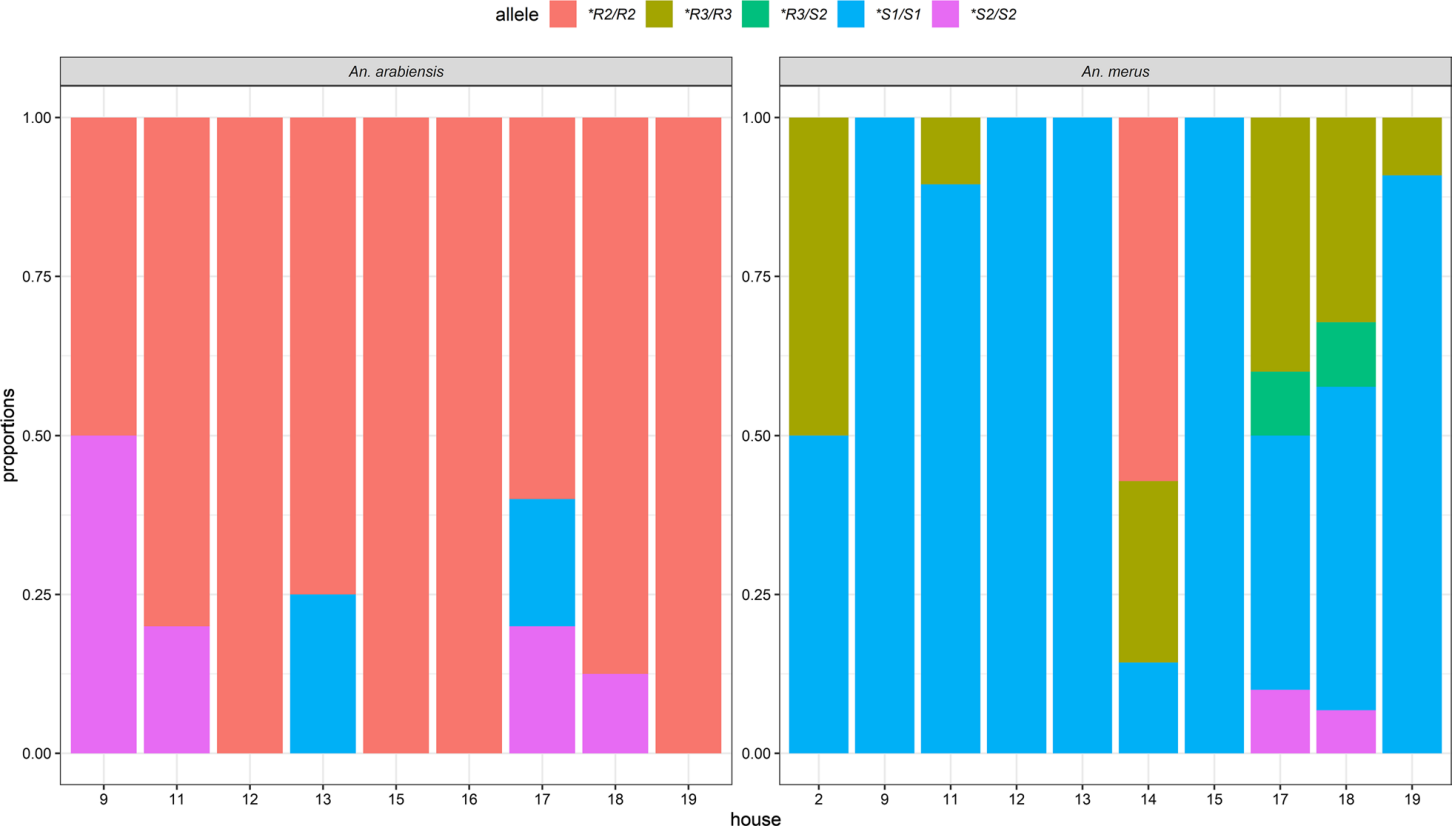
Proportions of the *TEP1* alleles found in the different houses in Garithe village along the Kenyan coast

We genotyped 175 samples from both *An. merus* and *An. arabiensis*. The *R2/R2 (84%, *n* = 32) allele was predominant in *An. arabiensis*, followed by *S2/S2 (11%, *n* = 4) (Fig. 2). For *An. merus*, the predominant allele was *S1/S1 (66%, *n* = 91), followed by *R3/R3 (22%, *n* = 30) (Fig. 2). At the household level, the *R2/R2 allele among *An. arabiensis* and *S1/S1 among *An. merus* were common for all the houses that were analysed (Fig. 3). Additionally, *R3/R3 and *R3/S2 genotypes were restricted to *An. merus* mosquitoes. In further analysis between *TEP1* alleles and *P. falciparum* positivity, only *An. merus* and *An. arabiensis* with the *R2/R2 and *S1/S1 alleles were positive for *P. falciparum* sporozoites (Additional file 2: Table S1).

### Haplotype statistics

Full-length *TEP1*-TED sequences were used for haplotype statistics and test of neutrality (*n* = 110, length = 758 bp) (Table 3). The abundance of haplotypes among the specific alleles was highest in TEP1*S1. Haplotype diversity ranged from 0 to 0.999, with TEP1*S1 having the highest value (0.999) and TEP1*R3 with the lowest (Table 3).

**Table 3:** Haplotype statistics and tests of neutrality from full *TEP1-TED* sequences

Test	*TEP1* alleles			
	All alleles	TEP1*S1	TEP1*R2	TEP1*R3
No. sequences	110	48	54	8
Length (bp)	758	758	758	758
No. haplotypes	66	47	18	1
Haplotype diversity	0.890	0.999	0.583	0.000
No. variable sites	116	92	83	0
No. mutations	121	92	83	0
Nucleotide diversity	0.056	0.050	0.020	0.000
Tajima’s *D*	2.747	2.996	-0.555	NA
Fu and Li’s *D*	1.426	1.297	0.861	0.000
Fu and Li’s *F*	2.398	2.298	0.392	0.000

In terms of haplotype diversity, TEP1*S1 was the most diverse allele. The low nucleotide diversity values indicate that there is a modest difference within the alleles

### *TEP1* phylogenetics

**Fig. 4:**
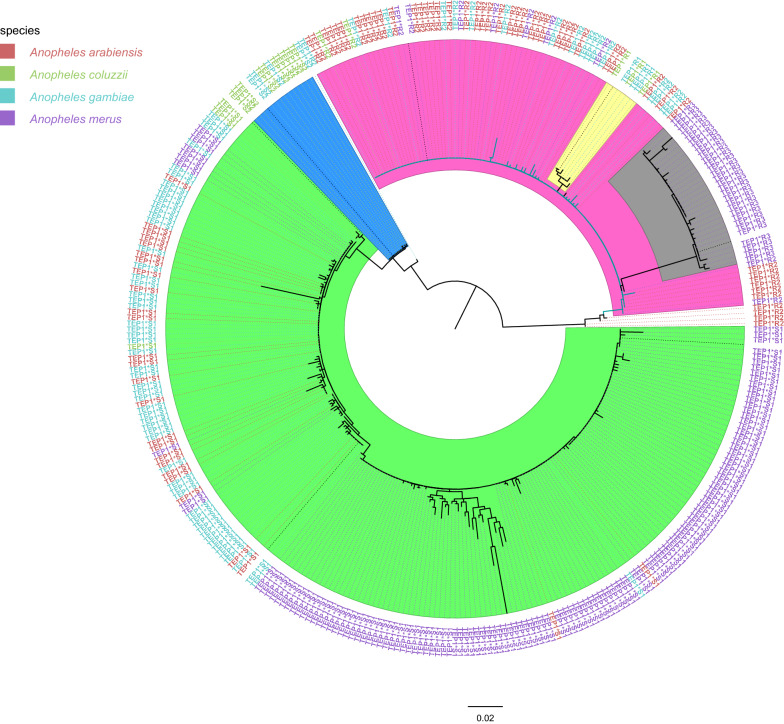
Maximum likelihood tree on the *TEP1-TED* domain of sequences from Garithe as well as GenBank. There are two clades: the susceptible (TEP1*S) and refractory (TEP1*R). Among the susceptible, two subclades, TEP1*S1 (green) and TEP1*S2 (blue), appear to be evolving independently of each other. Among the refractory alleles, TEP1*R1 (yellow) and TEP1*R2 (pink) are closely related, with TEP1*R1 forming a subclade nested within TEP1*R2. TEP1*R3 (grey) is restricted to *An. merus*

Generally, two major clades exist, with the susceptible (TEP1*S) and resistant (TEP1*R) genotypes having evolved separately and independently of each other (Fig. 4). The distribution of these alleles was not mosquito species-specific except for TEP1*R3, which was only found among *An. merus* mosquitoes. Amongst the susceptible, two further subclades exist (TEP1*S1 and TEP1*S2) that are evolving independently of each other since last separating from their most recent common ancestor (MRCA). The TEP1*S1 allele is the most prevalent overall, and more diverse than TEP1*S2. Amongst the mosquitoes carrying the resistant allele, two subclades exist (TEP1*R1-TEP1*R2 and TEP1*R3). TEP1*R1 and TEP1*R2 are closely related, with the former forming a subclade nested within TEP1*R2.

## Discussion

*Anopheles merus* was the predominant mosquito from the *An. gambiae* complex, constituting 77.2% of those that were identified by PCR, which is consistent with previous studies in the same area [21, 22, 39]. In contrast to other studies, we did not find any *An. gambiae* s.s., and therefore we assume that they may have been cleared by interventions such as IRS and LLINs [9, 40]. We also found that the majority of the mosquitoes were outdoor feeders, which could also be due to pressure from insecticides, as previously observed in Tanzania among *An. funestus* mosquitoes [9]. Additionally, *An. arabiensis* and *An. merus* have been shown to be exophilic and zoophilic [22, 39, 41, 42].

*Anopheles merus* is an important vector of *P. falciparum* and *Wuchereria bancrofti*. This mosquito species is especially notorious for its exophilic tendencies. From the 18s rRNA PCR, we identified a *P. falciparum* infection rate of 9.5% (20/210) in *An. merus* and 8.6% (6/70) in *An. arabiensis*, with a majority of these being outdoor feeders, meaning they may be involved in outdoor malaria transmission. Of note, the blood-fed mosquitoes identified either indoor or outdoor exhibited relaxed feeding preference by drawing blood meals from a variety of vertebrate hosts including humans, goats, and bovines.

Of interest for the *TEP1* alleles analysed, the TEP1*S1/S1 was the most prevalent allele (66%) and the only allele positive for sporozoite infection (*n* = 6). We also found the TEP1*R3 allele exclusively in *An. merus*, though being sympatric with *An. arabiensis*, which is consistent with previous findings [20, 43]. However, the function of the TEP1*R3 allele is yet to be elucidated, although our data suggest that they may be associated with mosquito refractoriness to *Plasmodium* infection, since none of the genotyped TEP1*R3 mosquitoes were positive for *P. falciparum*. The TEP1*R3 allele was fairly well distributed in Garithe and found in about a third of sampled houses (Fig. 3). The *S1/S1 and *R3/R3 genotypes were enriched in *An. merus* similar to previous findings [20, 43]. The *R1/R1 allele is more efficient in parasite clearance than *R2/R2, with *S2/S2 and *S1/S1 being fully susceptible.

The tests on haplotype diversity showed that the most diverse allele was TEP1*S1, followed by TEP1*R2 (Table 3). The low nucleotide diversity values for all the alleles indicate that there is a modest difference among the alleles. Tajima’s *D* values were positive for TEP1*S1, indicating that there is a balancing selection, and negative for TEP1*R2, indicating a recent selective sweep. As for TEP1*R3, there is no evidence of selection. Lastly, the Fu and Li’s *D* and *F* statistics were calculated, where all the values were positive, ranging from 0 to 2.398, indicative of a few unique variants and an abundance of the present variants.

Phylogenetic analysis showed that the refractory and susceptible *TEP1* alleles emerged independently of each other, as shown in the phylogenetic tree (Fig. 4). They exist as separate clades, each clade evolving uniquely and exhibiting high diversity. The separate emergence and evolution of the refractory and susceptible alleles is likely due to two different gene conversions in the *TEP1* loci [18]. The resistant and susceptible alleles have previously been reported to be recombinants and under positive selection [18]. It is also clear that the TEP1*R3 is restricted to the saltwater breeding *An. merus* from the Kenyan coast: Kwale and Kilifi counties. Why the allele is present in these mosquitoes is still a mystery and yet to be elucidated.

## Conclusions

*Anopheles merus*, a saltwater breeding mosquito vector of malaria and lymphatic filariasis, was the dominant species of *An. gambiae* complex identified in Garithe village in coastal Kenya. Mosquitoes in the study area were predominantly exophilic and utilised a variety of vertebrate hosts for blood meal requirements. Our observation of the abundance of the *P. falciparum* fully susceptible TEP1*S1 allele shows that the area has a huge number of mosquitoes that are ready to transmit malaria. This is evidenced by the high number of sporozoite-positive mosquitoes with the *S1/S1 genotype among *An. merus* mosquitoes. In addition, TEP1*S1 mosquitoes were predominantly exophilic, meaning that indoor adult mosquito control strategies may be ineffective for their control. This fact might suggest a potential risk of these mosquitoes becoming major players in persistent outdoor malaria transmission. Mosquitoes bearing the TEP1*R3 allele were the second most frequently found, and although the role of this allele in malaria has not been established, it is unlikely that they would support malaria given they were negative for *P. falciparum* infection and the fact that they belong to a family of refractory alleles. However, this needs further confirmation by experimental infections. Of interest the TEP1*R3 allele seems to be mosquito species-specific i.e. only found in *An. merus* that were also negative for *P. falciparum* infection. This obersavation suggests the TEP1*R3 allele has potential for consideration as candidate molecule for malaria transmission blocking through applictions such as gene drive systems.

## Supplementary Information

**Additional file 1: Figure S1.** The respective houses in Garithe village where mosquitoes were sampled.

**Additional file 1: Table S1.** The *P. falciparum* sporozoite rates between the different *TEP1* genotypes among *An. merus* and *An. arabiensis* mosquitoes.

**Additional file 3: Table S2.** The sporozoite rates between indoor and outdoor collected mosquitoes.

## Data Availability

The data and the analysis codes used in R (v 4.0.1) are available in the Harvard Dataverse: 10.7910/DVN/CKMX29. The sequences have been deposited at GenBank under accession numbers OM237458–OM237611.

## Abbreviations

DNA: Deoxyribonucleic acid
DnaSP: DNA Sequence Polymorphism software
ELISA: Enzyme-linked immunosorbent assay
KWTRP: KEMRI Wellcome Trust Research Programme
IgG: Immunoglobulin G
IRS: Indoor residual spraying
ITS: Intergenic spacer
LLINs: Long-lasting insecticide-treated nets
NGS: Next-generation sequencing
PBS: Phosphate-buffered saline
PCR: Polymerase chain reaction
*TEP1**R: *TEP1*-refractory
RT-PCR: Real-time polymerase chain reaction
*TEP1**S: *TEP1*-susceptible
TED: Thioester domain
*TEP1*: Thioester-containing protein 1
WHO: World Health Organization
