# Supplementary figures and images for: Vectorial capacity and TEP1 genotypes of Anopheles gambiae sensu lato mosquitoes on the Kenyan coast
Source: Parasit Vectors. 2022 Dec 1;15:448. doi: 10.1186/s13071-022-05491-5 (PMC9713959; doi:10.1186/s13071-022-05491-5)

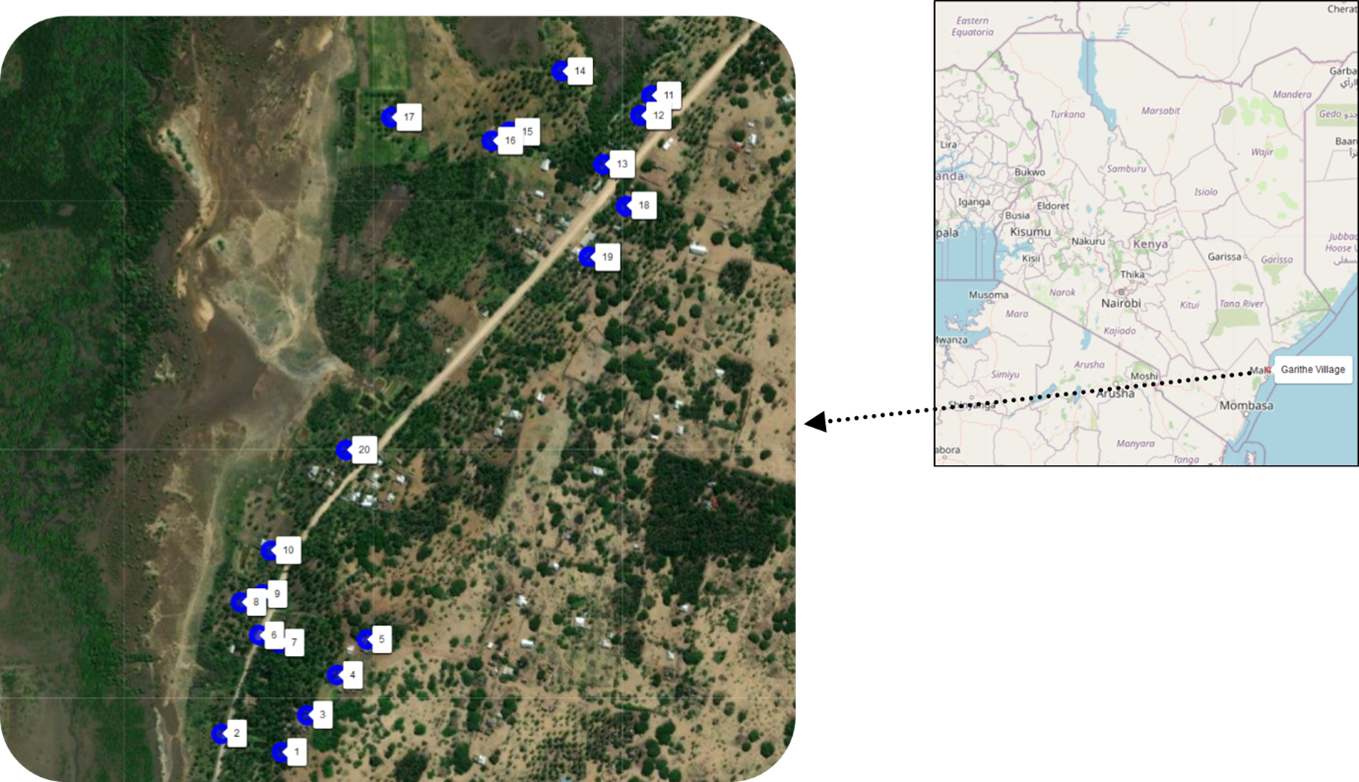

Supplement: Supplementary file 1 — Additional file 1: Figure S1. The respective houses in Garithe village where mosquitoes were sampled. [file 13071_2022_5491_MOESM1_ESM.docx]
