# Supplementary material for: Vectorial capacity and TEP1 genotypes of Anopheles gambiae sensu lato mosquitoes on the Kenyan coast
Source: Parasit Vectors. 2022 Dec 1;15:448. doi: 10.1186/s13071-022-05491-5 (PMC9713959; doi:10.1186/s13071-022-05491-5)
Supplement: Supplementary file 2 — Additional file 1: Table S1. The P. falciparum sporozoite rates between the different TEP1 genotypes among An. merus and An. arabiensis mosquitoes. [file 13071_2022_5491_MOESM2_ESM.docx]

| **species** | **allele** | **Negative (%)** | **Positive (%)** |
| --- | --- | --- | --- |
| *An. arabiensis* | *Not genotyped* | 48 | 0 |
|  | **R2/R2* | 14 | 5 |
|  | **S1/S1* | 0 | 1 |
|  | **S2/S2* | 2 |  |
| **Total** |  | **64 (91.4)** | **6 (8.6)** |
| *An. merus* | *Not genotyped* | 146 | 0 |
|  | **R2/R2* | 1 | 3 |
|  | **R3/R3* | 6 | 0 |
|  | **S1/S1* | 38 | 17 |
| **Total** |  | **191 (90.5)** | **20 (9.5)** |
