# Supplementary material for: Vectorial capacity and TEP1 genotypes of Anopheles gambiae sensu lato mosquitoes on the Kenyan coast
Source: Parasit Vectors. 2022 Dec 1;15:448. doi: 10.1186/s13071-022-05491-5 (PMC9713959; doi:10.1186/s13071-022-05491-5)
Supplement: Supplementary file 3 — Additional file 3: Table S2. The sporozoite rates between indoor and outdoor collected mosquitoes. [file 13071_2022_5491_MOESM3_ESM.docx]

| **species** | **site** | **sporozoite** | **Number of mosquitoes (%)** |
| --- | --- | --- | --- |
| *An. arabiensis* | Indoor | negative | 8 (88.9) |
|  |  | positive | 1 (11.1) |
|  | Outdoor | negative | 56 (91.8) |
|  |  | positive | 5 (8.2) |
| *An. merus* | Indoor | negative | 12 (92.3) |
|  |  | positive | 1 (7.7) |
|  | Outdoor | negative | 179 (90.4) |
|  |  | positive | 19 (9.6) |
